# Dose-Dependent Anti-Erosive Effect of Green Tea Extract Modification of Rivella Beverage

**DOI:** 10.3290/j.ohpd.c_2430

**Published:** 2026-01-28

**Authors:** Nicolai Blatter, Blend Hamza, Florian J. Wegehaupt

**Affiliations:** a Nicolai Blatter Doctoral Student in Dentistry, Clinic of Conservative and Preventive Dentistry, Center for Dentistry, University of Zürich, Zürich, Switzerland. Conducted the experiment as part of his dissertation and wrote the manuscript.; b Blend Hamza Assistant Physician, Clinic of Orthodontics and Pediatric Dentistry, Center for Dentistry, University of Zürich, Zürich, Switzerland. Critically reviewed the manuscript, supervised the project.; c Florian Just Wegehaupt Professor, Head of the Clinic of Conservative and Preventive Dentistry, Center for Dentistry, University of Zürich, Zürich, Switzerland. Conceived and designed the experiment, critically reviewed the manuscript, and supervised the study.

**Keywords:** erosive tooth wear, green tea, profilometry, soft drink.

## Abstract

**Purpose:**

To examine the effect of adding increasing amounts of green tea extract (GTE) to a soft drink (Rivella) on dentin wear in an erosion-only model.

**Materials and Methods:**

The study consists of two experiments: In the first experiment, a total of 60 bovine dentin samples from 4 groups (n=15) were immersed in four Rivella variants: red, blue, green, and yellow. The samples were subjected to 4 cycles (per cycle: 10 min in the specified solution followed by rinsing with deionised water for 5 sec then storage 60 min in artificial saliva). In the second experiment, a total of 120 bovine dentin samples from 8 groups (n=15) were immersed in Rivella red with modified GTE concentrations (0.0; 0.05, 0.2, 0.4, 0.8, 1.0 or 1.2%). The cycle procedure was the same as in the first experiment.  The measured dentin loss corresponded to the vertical shift on the y-axis between the baseline and the final profile after the wear process in 2D. Erosive dentin wear was measured by a stylus profilometer (µm). Data were analyzed using ANOVA followed by post-hoc pairwise comparisons and the p-values were adjusted after Holm.

**Results:**

In experiment 1 the following dentin wear (mean ± SD) was observed: Rivella red: 2.7 ± 0.4 µm; Rivella blue: 3.1 ± 0.4 µm; Rivella green: 2.1 ± 0.4 µm; Rivella yellow: 2.1 ± 0.3 µm. While the first two differed significantly, the last two did not.

In experiment 2 dentin wear (mean ± SD) was: Rivella+0% GTE: 3.6 ± 0.6) µm; Rivella+0.05% GTE: 3.6 ± 0.2) µm; Rivella+0.2% GTE: 3.4 ± 0.6) µm; Rivella+0.4% GTE: 3.1 ± 0.4) µm; Rivella+0.6% GTE: 2.6 ± 0.3) µm; Rivella+0.8% GTE: 2.6 ± 0.4) µm; Rivella+1.0% GTE: 2.2 ± 0.2) µm; Rivella+1.2% GTE: 2.1 ± 0.3) µm. A significant decrease in erosive wear with increasing amount of GTE was observed.

**Conclusion:**

When increasing the addition of green tea extract to the soft drink Rivella, an almost linearly increasing protection against erosion can be observed in vitro.

Distinct from dental caries, there is an increasing prevalence of dental hard tissue loss that is not primarily caused by bacterial activity. This phenomenon encompasses both mechanical and chemical processes and is collectively referred to as tooth wear. Mechanically induced wear includes attrition, which results from tooth-to-tooth contact, and abrasion, which is caused by external mechanical factors such as toothbrushing or abrasive substances. In addition, erosion represents a chemically induced loss of hard dental tissues due to the action of non-bacterial acids. Dental wear, particularly erosive tooth wear, has emerged as a growing clinical and epidemiological challenge, requiring careful diagnosis and management.

Erosion is a specific type of tooth wear and can be a possible pathological cause.^26^ Acid attacks^15,27^ can be of external origin through exposure or consumption of acidic substances (e.g., acidic foods, medicines and industrial acids) which lead to increased surface erosion, particularly on the occlusal surface and the buccal aspects of the teeth.^25^ On the other hand, it may be that the acid effect is caused by intrinsic factors, as in the case of regurgitation of gastric acid into the oral cavity (e.g., reflux), which lead to increased erosive washouts on the lingual aspects of the teeth.^3^ The increasing consumption of acidic soft drinks is a significant factor causing tooth erosion and has already been investigated in several studies.^9,21,23^

It has been shown that the addition of certain substances to soft drinks can have a prophylactic effect and help reduce erosive wear on the teeth.^14^ An important finding was that not only the pH but also the buffering capacity is crucial for the erosive potential of a beverage.^26^ For example, it was shown that beverages fortified with calcium, fluoride and phosphate had a reduced erosive potential.^18^ A lower pH alone is not enough to cause significant erosion.^26^ An essential role in the erosion process of dentin can be attributed to the matrix metalloproteinases (MMPs) and have already been investigated by several in-vitro and in-situ studies.^6,24^These enzymes are present in dentin and saliva;^1,5,20^ their function is to degrade demineralized dentin. The collagen fibrils, which are initially exposed in the dentin by the low pH of the erosion agent, are vulnerable to MMPs.^16^ As soon as the pH returns to neutral, the MMPs resume degrading the demineralized organic matrix, furthering the loss of dentin.^5^ Inhibition of these enzymes can therefore reduce dentin erosion. Green tea polyphenols, in particular epigallocatechin-3-gallate (EGCG), have been shown to inhibit these enzymes in various studies.^2,4,11^ It changes the organic matrix in such a way that the activity of MMP2 and MMP9, which are essential for the degradation process, is inhibited.^8^ Rivella Green (Rivella AG; Rothrist, Switzerland), a drink established on the Swiss market, already contains 0.05% of the green tea extract mentioned. Rivella is one of the most popular drinks in Switzerland. In 2013, Rivella AG achieved a market share of 15.3% in the Swiss soft-drinks sector. This puts Rivella in second place behind Coca-Cola and has accounted for almost one-sixth of all drinks sold in recent years. This in-vitro study was conducted to investigate (1) the question of whether the other Rivella variants differ in their erosive potential and (2) which concentration of green tea extract was necessary in the most widely-consumed Rivella variant (Rivella red) to significantly reduce the erosive potential.

The null hypothesis was that there is no statistically significant difference in erosive wear between the Rivella variants (red, blue, green, and yellow) or in Rivella red with the addition of green tea extract.

## MATERIALS AND METHODS 

The study was divided into two experiments: Experiment I (determination of erosive potential of Rivella variants red, blue, green and yellow) and Experiment II (reduction of erosive potential by adding green tea extract to the oldest and most frequently consumed Rivella variant, Rivella red).

### Preparation of the Samples for Both Experiments

A total of 180 dentin samples were prepared for both experiments. Sixty samples were used for Experiment I and 120 samples for Experiment II. The dentin samples were prepared from bovine mandibular anterior teeth obtained from a local slaughterhouse; the animals had previously been intended for meat consumption and were unrelated to the present study. The samples were prepared from several teeth and randomly distributed between the two experiments. For obtaining pure dentin, the roots of extracted bovine teeth were used. The superficial root cementum was completely ground away and polished during the preparation process with carborundum paper (SiC paper, Struers; Birmensdorf, Switzerland). The preparation steps were performed identically for all samples as follows: using a Trepan diamond drill with an inner diameter of 3 mm (Turning & Milling Machine System, PROXXON; Föhren, Germany), the samples (cores) were drilled out from the roots under simultaneous water cooling. This was followed by embedding the dentin cores in acrylic resin (Paladur, Heraeus Kulzer; Hanau, Germany), whereby the total diameter of the sample was increased to 6 mm. Polymerization at 45°C and 2 bar was achieved in 10 min using a polymerization device (Palamat elite, Heraeus Kulzer). The side of the sample to be examined, which was exposed to the test solutions in the experiment, was ground and polished. The grinding process, which consisted of two phases, was carried out with a grinding device (Tegramin-30, Struers). First, the samples were clamped in the machine and ground with a carborundum paper with a grit size of 2000 for 15 s and a contact pressure of 5 N. As already mentioned, the root cement was removed. To further polish the surface, a 4000-grit carborundum paper was used with a running time of 30 s and the same contact pressure of 5 N. Second, two parallel lines were carved into each sample using a specially manufactured device (in-house production, Conservative and Preventive Dentistry, Center for Dental Medicine, University of Zürich, Switzerland). The scratches on both the acrylic resin and the dentin were made using a sharp metal tip attached to the apparatus. Each sample was clamped into the specially manufactured device in the same position and orientation, so that standardised reference scratch marks could be produced. These scratches were used as a reference structure during the later profilometric measurement. The sample had to be protected from the erosive solution so that both the orientation scratches and part of the dentin were covered with adhesive tape (Scotch Crystal Tape 600, 3M; Rüschlikon, Switzerland). Care was taken to ensure that a residual 2-mm dentin strip was uncovered and could therefore be exposed to the test solutions. During later allocation of the samples to the different groups, it was ensured that not more than one sample of a single root was allocated to one group.

### Experiment I: Determining the Erosive Potential of Various Types of Rivella

Sixty (60) samples were divided into four test groups: Rivella red (91 ppm calcium, 70 ppm phosphorus, pH = 3.21); Rivella blue (122 ppm calcium, 72 ppm phosphorus, pH = 3.22); Rivella green (68 ppm calcium, 70 ppm phosphorus, pH = 3.24, 0.05% green tea extract); Rivella yellow (87 ppm calcium, 0 ppm phosphorus, pH = 3.13). The sample size was chosen to resemble similar erosion studies investigating the effect of green tea extract.^4,10^ Erosive demineralization was carried out in four cycles, which were completed in one day. In each cycle, the samples were first exposed to the test solution for 10 min at 25°C. For this purpose, the samples of the same groups were placed in a common sample container. The samples themselves were attached to a specially made holder which could be immersed precisely into the sample containers holding the test solution. After the samples were immersed in the solution, they were left undisturbed for 10 min to allow the erosion process to take place.

For each immersed sample, 8 ml of the solution was added to the container using a ml-graduated pipette. The solution was always pipetted into the corner of the container.

After each erosive attack, the samples were rinsed with deionized water. The samples were then stored in artificial saliva at 37°C for 60 min. This process was repeated four times in total for all dentin samples. After opening the drinks, the first cycle had to be started immediately. The drinks were sealed airtight during each cycle. After opening the drinks, the beverages were used for all four cycles for 4 h 50 min. Table 1 shows the main ingredients of all four Rivella products used in Experiment I.

**Table 1:** Ingredients of Rivella products

Variant	Main ingredients	Sweetener	Special additions
Rivella red	Water, milk whey (35%), carbonic acid (CO_2_), acidity regulator (lactic acid), caramelized sugar, natural flavorings	Sugar 25%	–
Rivella blue	Water, milk whey, carbonic acid, acidity regulator (lactic acid), caramelized sugar, natural flavorings	Sugar 4% Artificial sweeteners (cyclamate, acesulfame K)	–
Rivella green	Water, milk whey, carbonic acid, acidity regulator (lactic acid), caramelized sugar, natural flavorings	Sugar 14%	Green tea extract (0.05%), barley malt extract, vitamin C
Rivella yellow	Water, lactose-free milk whey, carbonic acid, acidity regulator (malic acid), caramelized sugar, natural flavorings	Sugar 5.2%	–


### Experiment II: Determining the Effect of Supplementing Rivella Red with Various Concentrations of Green Tea Extract 

The remaining 120 samples were divided into the following eight test groups according to the amount of the supplemented green tea extract: group 1: no supplementation; group 2: 0.05% green tea extract (OM24, Omnimedica; Schlieren, Switzerland); group 3: 0.2% green tea extract; group 4: 0.4% green tea extract; group 5: 0.6% green tea extract; group 6: 0.8% green tea extract; group 7: 1.0% green tea extract; group 8: 1.2% green tea extract. The precisely calculated amount of green tea extract added to each test solution was weighed using a suitable balance (Top-loading balance TM300, Mettler Toledo; Greifensee, Switzerland). The mixing of the extract with the test solution was then carried out using a magnetic stirrer (Hotplate Stirrers, IKA; Staufen, Germany). In Experiment II, the samples underwent the same erosive cycling as in Experiment I.

### Determining Dentin Wear

To determine the erosive dentin wear, a baseline profile was taken before the erosive attack. This recording was performed with a stylus profilometer (Perthometer S2, Mahr; Göttingen, Germany). After the erosive attacks on the dentin samples had been completed, the final profiles and thus the final measurement profiles were created directly. The loss of tooth substance on the dentin surface caused by the solution resulted in a measurable vertical height (y-axis) difference before and after erosion.

For the baseline and final profiles, five parallel measurement lines were recorded at a distance of 250 µm and a length of 4.8 mm. A special device was used to precisely position the samples in the profilometer when recording the initial measurement and reposition them when measuring the final profile. The initial and final profiles were compared by superimposing them at this point. The accuracy of the profilometric measurements was 40 nm (in the y-axis), as in the preliminary studies.^4,11^ Under experimental settings, the minimum detection threshold of the profilometer is 100 nm. All data were collected using specially developed computer software (4D Client, specially adapted, Conservative and Preventive Dentistry, Center for Dental Medicine, University of Zürich, Switzerland). The comparison of the baseline and profiles allowed measuring the vertical change of each sample’s dentin surface. Tables 2 and 3 provide an overview of the research design for Experiments I and II.

### Statistical Analysis Experiments I and II

Data normality was evaluated using Shapiro–Wilk tests and inspection of Q–Q plots of group-wise outcomes and model residuals. Homogeneity of variances was verified by visual assessment of residual vs fitted plots. As no statistically significant deviations from normality were detected, parametric analyses (ANOVA and linear regression) were applied. Mean and standard deviation (SD) of the erosive dentin wear in the experimental groups were calculated. Pairwise comparisons between the groups (within Experiment I and Experiment II separately) were carried out using ANOVA and Tukey’s HSD post-hoc test. p-values were adjusted for multiple testing using Holm’s method correction. All data were evaluated with ANOVA and analyzed with the statistical program R (The R Foundation for Statistical Computing; Vienna, Austria; www.R-project.org). Additionally, a regression was determined. The y-axis shows the erosive removal in µm in relation to the added amount of green tea extract on the x-axis. A red linear trend was included in the boxplot to provide a visual impression of a potential linear relationship of the data. This linear trend was determined by linear regression. For comparison, a gray line was included that connects the median of each group.

## RESULTS

### Experiment I

**Fig 1:**
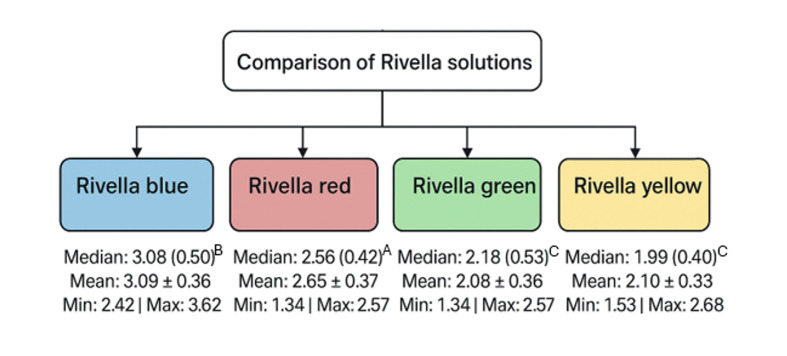
Erosive dentin wear (in µm). Different superscript letters indicate a statistically significant difference between the groups.

Figure 1 shows the resulting erosive wear in each group of Experiment I. The mean and standard deviation (SD) values for erosive dentin wear were as follows: Rivella blue: 3.1 µm (0.4); Rivella red: 2.7 µm (0.4); Rivella green: 2.1 µm (0.4); Rivella yellow: 2.1 µm (0.3). The comparison between all the groups tested proved to be statistically significant (p < 0.05), with the exception of the comparison between Rivella green and Rivella yellow (p = 0.8). Wear with sugar-free Rivella blue was statistically significantly higher compared to that of the most commonly consumed variety, Rivella red, which contains sugar (p = 0.0028). When comparing Rivella red to Rivella yellow, the difference was once again statistically significant, and the same applied to the comparison of Rivella red to Rivella green (both p = 0.0002).

### Experiment II 

**Fig 2:**
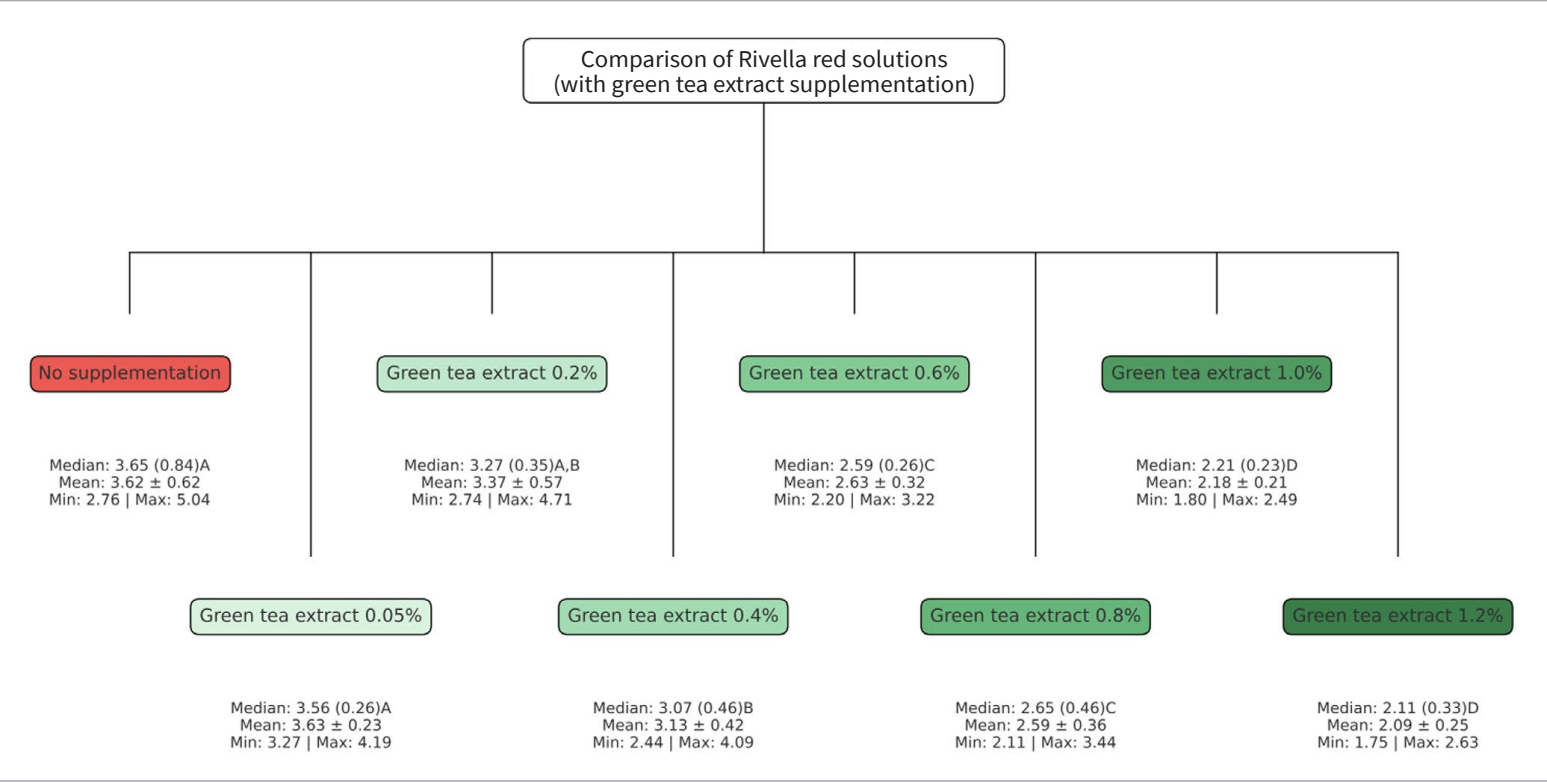
Erosive dentin wear (in µm). Different letters indicate a statistically significant difference between the groups.

Figure 2 shows the resulting erosive wear in each experimental group of Experiment II. The means ± SD of erosive dentin wear were as follows: Rivella red: 3.6 µm (0.6); Rivella red with green tea extract 0.05%: 3.6 µm (0.2); Rivella red with green tea extract 0.2%: 3.4 µm (0.6); Rivella red with green tea extract 0.4%: 3.1 µm (0.4); Rivella red with green tea extract 0.6%: 2.6 µm (0.3); Rivella red with green tea extract 0.8%: 2.6 µm (0.4); Rivella red with green tea extract 1.0%: 2.2 µm (0.2); Rivella red with green tea extract 1.2%: 2.1 µm (0.3). When comparing the groups with each other, no statistically significant difference in wear was observed between the unmodified solution and the first two test solutions with 0.05% (p = 1.0009) and 0.2% (p = 0.4652) green tea extract. A statistically significant difference could be seen for when 0.4% green tea extract was added to the unmodified solution (p = 0.0092). Between all other test solutions, statistically significant differences were found. Exceptions were the comparison between the test solution with 0.05% and the next higher concentration of 0.2% green tea extract (p = 0.4652). The same applies to the comparison between 0.2% and 0.4% green tea extract (p = 0.4652), 0.6 and 0.8% (p = 1.0000) and 1.0 and 1.2% (p = 1.0000).

## DISCUSSION

The aim of this study was to show in a preliminary experiment that different variants of a soft drink of the same brand, which differ in pH and ingredients (in particular in the type of acid), differ in their erosion potential. The second part was to show which concentration of added green tea extract is required to achieve a statistically significant reduction in the erosion potential of the soft drink under investigation. As a statistically significant difference was observed in both experiments in this study, the null hypothesis was rejected.

As in many other studies, bovine teeth were also used in this investigation. Compared to human teeth, they show no statistically significant difference in terms of erosion resistance.^2,4,11,21^

Contact stylus profilometry represents a well-established, robust, and highly reproducible technique for quantifying erosive tissue loss on hard dental substrates. Its main advantage lies in its high vertical sensitivity and precision, allowing reliable detection of micrometric changes in surface height. Because the method provides absolute vertical displacement between reference and eroded areas, it is particularly suitable for standardized in-vitro studies on polished enamel or dentin surfaces. However, being a contact-based measurement, the diamond stylus can theoretically cause minute surface scratching, although previous work has shown that the resulting artifacts are negligible when low contact forces (≤1 mN) and fine stylus tips (about 2 µm) are used. In addition, profilometry captures only linear profiles and requires flat, smooth surfaces, which may limit its applicability to complex three-dimensional or rough specimens. Despite these limitations, contact stylus profilometry remains a valid and precise method for quantifying erosive dentin loss when surfaces are properly prepared and measurement parameters are carefully standardized.^10^ In contrast, non-contact profilometric methods, such as focus variation 3D, are non-destructive, provide three-dimensional surface data, and eliminate any risk of surface damage. These optical techniques are also faster and can capture complex surface topographies, making them potentially superior for assessing irregular or curved specimens.

**Fig 3:**
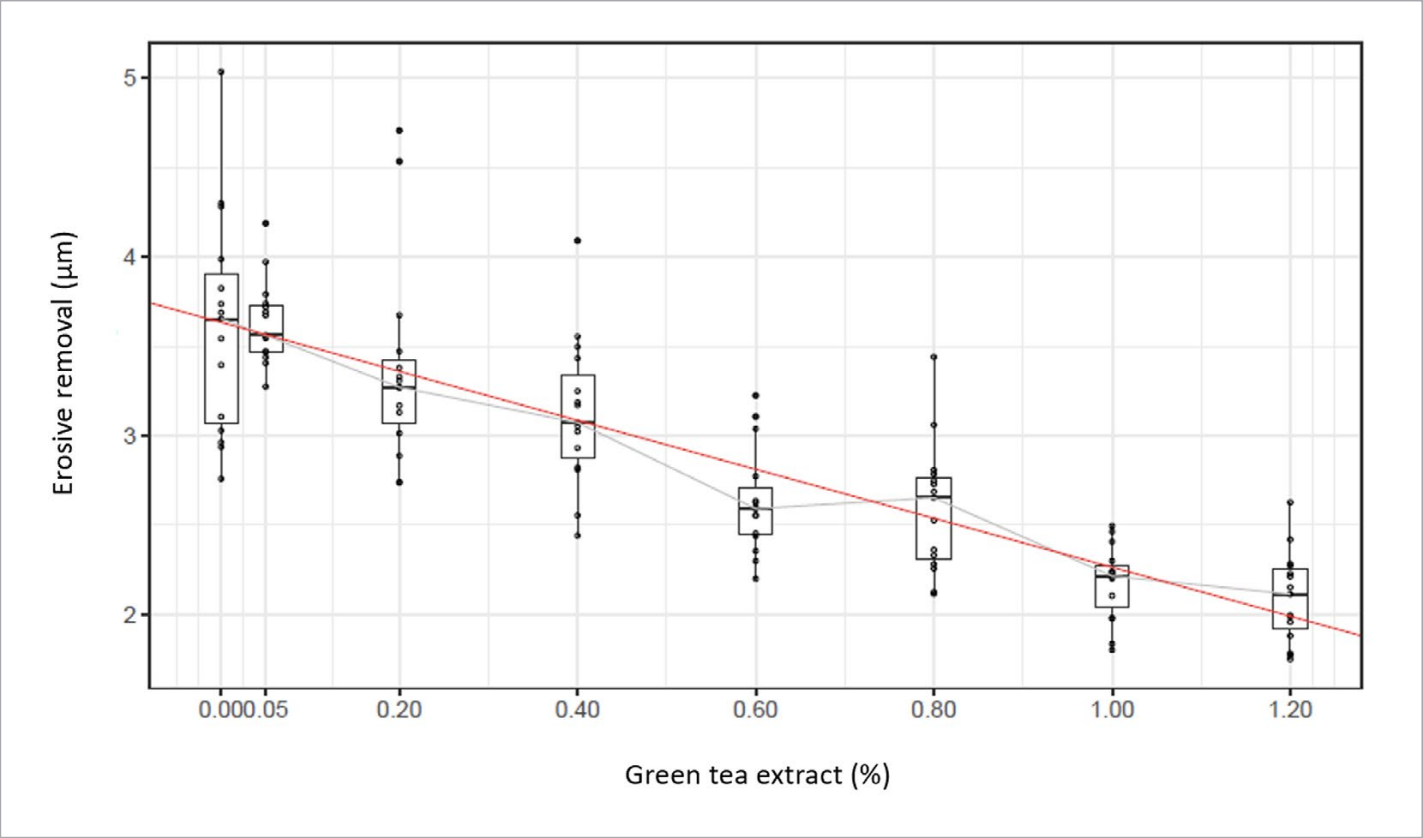
Linear regression of the erosive dentin wear with increasing concentration of green tea extract.

It is known that the chemical process of erosion depends on the pH, acid concentration, and the buffering capacity of the solution used.^17,22^ For example, it has been shown that a low pH and a high concentration of acids lead to the most intense erosion, while a high pH and low acid concentration resulted in the least erosion. It has also been shown that different types of acid cause different degrees of erosion. At low pH values, for example, lactic acid was more erosive than citric or malic acid.^13^ This can also be observed in the present study where, in the preliminary test, Rivella yellow, which contains malic acid, is statistically significantly less erosive than Rivella red, which contains lactic acid, although the pH of Rivella yellow is almost the same or even 0.08 pH units lower than that of Rivella red. Surprisingly, the sugar-free variant Rivella Blue produces statistically significantly greater erosion than the sugar-containing variant Rivella Red. This can also be observed in the study by Hamza et al,^11^ where the sugar-free energy drink has a slightly higher erosivity than the sugar-containing one. Furthermore, it can be seen that there is a statistically significant difference between Rivella Green with added green tea extract and Rivella Red without, and that the erosion is also smaller here. Interestingly, the added green tea extract amounts to just 0.05%. It should be noted that other additional ingredients, e.g., barley malt extract, have been added to Rivella Green, which could also have an influence. We found that in the second experiment, the addition of green tea extract to Rivella Red in stages also resulted in a gradual decrease in tooth wear. The amount of erosive dentin wear correlated with the added concentration of green tea extract formed an almost linear regression (Fig 3). With higher concentrations of added green tea extract, a slightly higher pH was also measured, which plays an essential role in erosion as described. The pH values by increasing concentration of green tea extract were: group 1: no supplementation (pH = 3.21); group 2: 0.05% green tea extract (pH = 3.25); group 3: 0.2% green tea extract (pH = 3.30); group 4: 0.4% green tea extract (pH = 3.36); group 5: 0.6% green tea extract (pH = 3.42); group 6: 0.8% green tea extract (pH = 3.46); group 7: 1.0% green tea extract (pH = 3.50) and group 8: 1.2% green tea extract (pH = 3.54). It should be noted that a statistically significant difference from the unmodified beverage was only detectable from an addition of 0.4% green tea extract onwards. Thus, it can also be stated that not only the green tea extract in Rivella Green is the reason for the statistically significant difference in erosion between Rivella Red and Rivella Green, but also the other added ingredients, such as the barley malt extract mentioned. In principle, a linear decrease in erosive wear can be observed by adding green tea extract. In research, green tea extract has also been shown to play a crucial role in reducing tooth erosion in several other studies.^7,12,19^

The main limitations of this study include the use of bovine dentin instead of human enamel, the in-vitro nature of the experiments, and the restriction to a single soft drink brand. As mentioned above, while bovine dentin has comparable erosive behavior to human hard tissues, future studies should validate these results on human enamel to improve clinical relevance. Additionally, the short-term erosive protocol used here does not fully replicate intraoral conditions, where factors such as pellicle formation, saliva buffering, and the presence of abrasive challenges would alter the outcomes. Future work should therefore explore the interaction between green tea extract concentration, acid/beverage type, and exposure dynamics on human enamel using non-contact profilometry and in-situ or in-vivo models. Such studies could also investigate whether the observed protective effect remains stable over time and in the presence of biofilm and salivary components.

## CONCLUSIONS

The type and concentration of acid as well as the concentration of added ingredients such as green tea extract had a reducing effect on the erosive potential of the soft drink under investigation. Furthermore, it was found that the concentration and stepwise increase of added green tea extract had a nearly linear regression in correlation to the reduction of erosive wear.

## ACKNOWLEDGEMENTS

The green tea extract was supplied by OM24, Omnimedica, Schlieren, Switzerland. The company had no influence on the study design, conducting the study, data collection and analysis, or the preparation of the manuscript.

**Table 3:** Design for Experiment II

Preparation of 120 dentin samples from bovine mandibular anterior teeth
**Randomized allocation of the samples in 8 groups (n = 15)**
Group 1 Rivella red	Group 2 Rivella red + 0.05% green tea extract	Group 3 Rivella red + 0.2% green tea extract	Group 4 Rivella red + 0.4% green tea extract	Group 5 Rivella red + 0.6% green tea extract	Group 6 Rivella red + 0.8% green tea extract	Group 7 Rivella red + 1.0% green tea extract	Group 8 Rivella red + 1.2% green tea extract
Recording of baseline profiles
4 cycles
Demineralization in pure beverage for 10 min (8 ml per sample) at 25°C
Rivella red	Rivella red + 0.05% green tea extract	Rivella red + 0.2% green tea extract	Rivella red + 0.4% green tea extract	Rivella red + 0.6% green tea extract	Rivella red + 0.8% green tea extract	Rivella red + 1.0% green tea extract	Rivella red + 1.2% green tea extract
Rinsing with deionized water (5 s)
Remineralization in artificial saliva for 60 min (8 ml per sample) at 37°C
Recording of final profiles


**Table 2:** Design for Experiment I

Preparation of 60 dentin samples from bovine mandibular anterior teeth
**Randomized allocation of the samples in 4 groups (n = 15)**
Group 1 Rivella red	Group 2 Rivella blue	Group 3 Rivella green	Group 4 Rivella yellow
Recording of baseline profiles
4 cycles
Demineralization in pure beverage for 10 min (8 ml per sample) at 25°C
Rivella red	Rivella blue	Rivella green	Rivella yellow
Rinsing with deionized water (5 s)
Remineralization in artificial saliva for 60 min (8 ml per sample) at 37°C
Recording of final profiles

